# Comparative proteomics combined with analyses of transgenic plants reveal ZmREM1.3 mediates maize resistance to southern corn rust

**DOI:** 10.1111/pbi.13129

**Published:** 2019-04-23

**Authors:** Shunxi Wang, Zan Chen, Lei Tian, Yezhang Ding, Jun Zhang, Jinlong Zhou, Ping Liu, Yanhui Chen, Liuji Wu

**Affiliations:** ^1^ Synergetic Innovation Center of Henan Grain Crops Henan Agricultural University Zhengzhou China; ^2^ Key Laboratory of Physiological Ecology and Genetic Improvement of Food Crops in Henan Province Zhengzhou China; ^3^ Section of Cell and Developmental Biology University of California at San Diego La Jolla CA USA; ^4^ Cereal Crop Research Institute Henan Academy of Agricultural Sciences Zhengzhou China

**Keywords:** Southern corn rust, maize, transgenic, remorin, proteomic, resistance

## Abstract

Southern corn rust (SCR), which is a destructive disease caused by *Puccinia polysora* Underw. (*P. polysora*), commonly occurs in warm‐temperate and tropical regions. To identify candidate proteins related to SCR resistance and characterize the molecular mechanisms underlying the maize–*P. polysora* interaction, a comparative proteomic analysis of susceptible and resistant maize lines was performed. Statistical analyses revealed 1489 differentially abundant proteins in the resistant line, as well as 1035 differentially abundant proteins in the susceptible line. After the *P. polysora* infection, the abundance of one remorin protein (ZmREM1.3) increased in the resistant genotype, but decreased in the susceptible genotype. Plant‐specific remorins are important for responses to microbial infections as well as plant signalling processes. In this study, transgenic maize plants overexpressing *ZmREM1.3* exhibited enhanced resistance to the biotrophic *P. polysora*. In contrast, homozygous *ZmREM1.3* UniformMu mutant plants were significantly more susceptible to *P. polysora* than wild‐type plants. Additionally, the *ZmREM1.3*‐overexpressing plants accumulated more salicylic acid (SA) and jasmonic acid (JA). Moreover, the expression levels of defence‐related genes were higher in *ZmREM1.3*‐overexpressing maize plants than in non‐transgenic control plants in response to the *P. polysora* infection. Overall, our results provide evidence that ZmREM1.3 positively regulates maize defences against *P. polysora* likely *via* SA/JA‐mediated defence signalling pathways. This study represents the first large‐scale proteomic analysis of the molecular mechanisms underlying the maize–*P. polysora* interaction. This is also the first report confirming the remorin protein family affects plant resistance to SCR.

## Introduction

Rust fungi constitute the largest group of fungal plant pathogens, with more than 7000 species described, some of which are responsible for three rusts diseases of corn [i.e. common rust, southern corn rust (SCR) and tropical rust] (Rochi *et al*., 2016). Southern corn rust, which is caused by *Puccinia polysora* Underw. (*P. polysora*), commonly occurs in warm‐temperate and tropical regions and has become one of the most destructive plant diseases in the United States of America (Raid *et al*., 1988), Asia (Liu *et al*., 2003) and Africa (Agarwal *et al*., 2001). Disease symptoms induced by *P. polysora* first appear on leaves, resulting in necrotic lesions, which then spread to the rest of the plant, ultimately damaging the photosynthetic activities and resulting in death (Cammack, 1958). Southern corn rust was detected in 1949 in West Africa, where it decreased corn yield by about 50% (Nattrass, 1953; Stanton and Cammack, 1953). In China, SCR was first observed in 1998, resulting in yield losses of 42%–53% (Zhou *et al*., 2007). Because of global warming, SCR has gradually spread to high‐latitude areas and has recently become a serious threat to crop production worldwide.

Southern corn rust and common rust are two major rust diseases affecting maize production, while tropical rust has been eradicated and has not been detected in the United States of America since 1970. Many quantitative trait loci associated with maize common rust resistance have been detected through genome‐wide association studies (Olukolu *et al*., 2016). Additionally, major resistance genes have been identified *via* forward and reverse genetic methods (Jamann *et al*., 2016; Sucher *et al*., 2017). However, SCR‐resistance genes have not been identified or cloned. Like other plant pathogenic microbes, *P. polysora* evolves rapidly and the associated wide dispersal of anemochorous spores increases the difficulty of controlling this fungus (Brewbaker *et al*., 2015). Moreover, the lack of relevant genetic information has prevented the complete characterization of the molecular mechanism underlying SCR resistance.

Proteomics, which refers to the global analysis of protein‐level changes, is useful for elucidating the mechanisms involved in maize–pathogen interactions, leading to the identification of defence‐ or resistance‐associated proteins (Pechanova and Pechan, 2015; Wu *et al*., 2013a,2013b, 2015a). In this study, iTRAQ as well as SCR‐resistant (P178) and SCR‐susceptible (Lx9801) maize inbred lines were used to identify proteins associated with maize resistance to *P. polysora*. A comparison proteomic analysis of SCR‐infected P178 and Lx9801 plants revealed that the abundance of one remorin protein, ZmREM1.3, was significantly greater in the SCR‐resistant genotype. Thus, this protein underwent a functional analysis based on genetic, biochemical and physiological experiments. This study may help to elucidate the interaction between maize and *P. polysora*, and lead to the development of new SCR‐resistant genetic resources applicable for maize breeding programmes.

## Results

### Phenotypic comparison of P178 and Lx9801 plants infected with *P**. **polysora*

**Figure 1:**
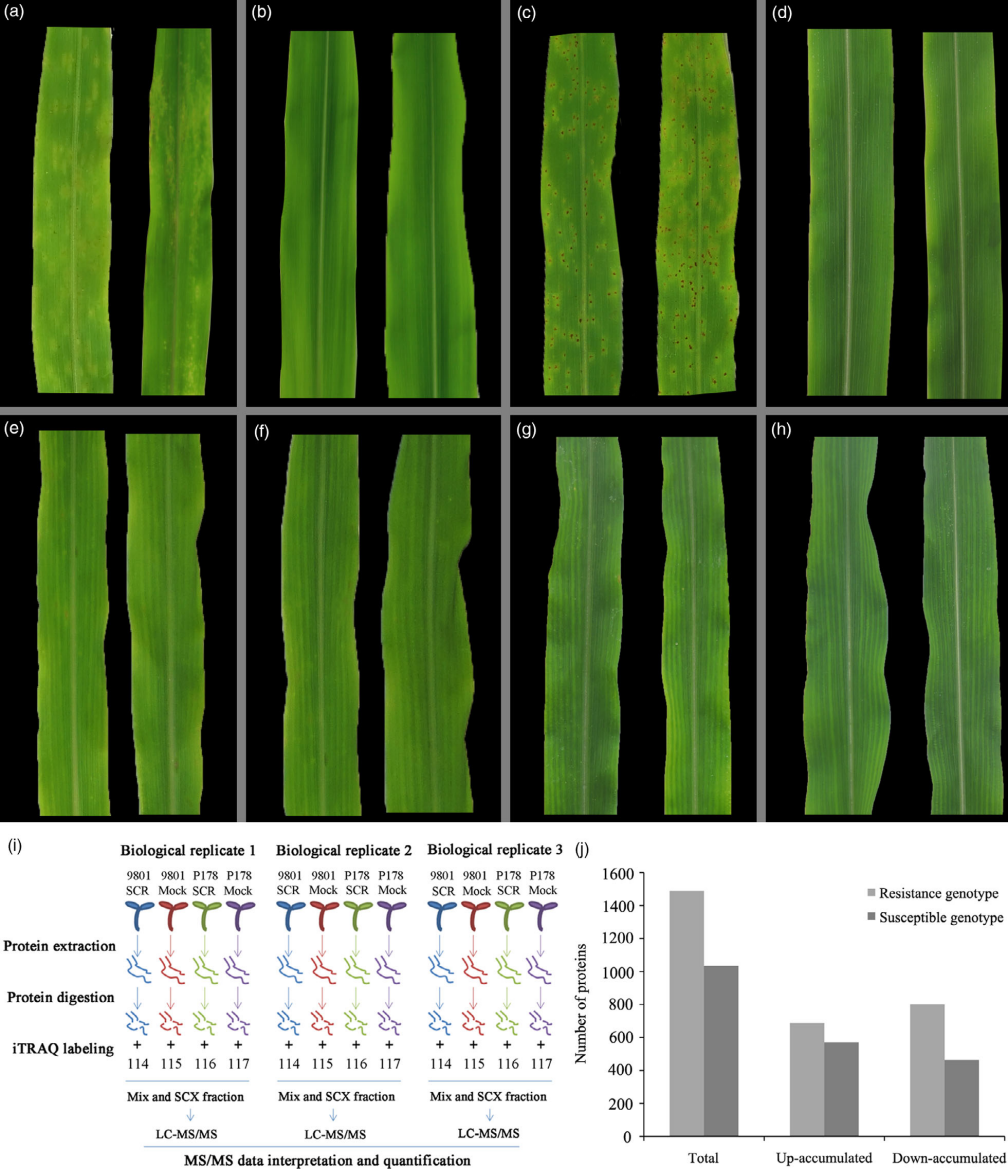
Phenotypic comparison of P178 and Lx9801 plants inoculated with *P. polysora*, iTRAQ workflow and distribution of differentially accumulated proteins. Phenotype of the susceptible line (Lx9801) plants at 7 days after the *P. polysora* (a) and mock (b) inoculations. Phenotype of the Lx9801 plants at 14 days after the *P. polysora* (c) and mock (d) inoculations. Phenotype of the resistant (P178) plants at 7 days after the *P. polysora* (e) and mock (f) inoculations. Phenotype of the P178 plants at 14 days after the *P. polysora* (g) and mock (h) inoculations. (i) iTRAQ 4‐plex Labelling and LC‐MS/MS workflow of identifying differentially accumulated proteins in both susceptible (Lx9801) and resistant (P178) lines after the *P. polysora* and mock inoculations. (j) Distribution of differentially accumulated proteins.

The phenotypes of two maize inbred lines, P178 (resistant line) and Lx9801 (susceptible line), were compared following *P. polysora* infection. At 7 days after inoculation, circular to oval and light‐orange pustules developed primarily on the surface of Lx9801 leaves (Figure 1a), while the mock‐inoculation plants were symptomless (Figure 1b). At 14 days after inoculation, the pustules on Lx9801 leaves erupted to expose dust‐like, gold spores (Figure 1c). In contrast, the mock‐inoculated plants were symptomless (Figure 1d). Additionally, P178 plants lacked the visible symptoms after the *P. polysora* or mock inoculations at all analysed time points (Figure 1e–h). The results showed that P178 is highly resistant to SCR and Lx9801 is highly susceptible to SCR.

### Identification of differentially accumulated proteins

To identify the proteins affected by the *P. polysora* infection, leaves of four sample groups (Lx9801SCR, Lx9801Mock, P178SCR and P178Mock) at 14 days after inoculation were collected. Total leaf proteins were extracted, and their profiles were investigated in a 4‐plex iTRAQ set; three independent biological replicates (three independent 4‐plex iTRAQ sets) were applied (Figure 1i). In total, 34 319 peptides corresponding to 6612 proteins were identified from 731 655 mass spectra with 95% confidence and a FDR ≤ 0.01 (Table S1). All mass spectral data associated with this study have been deposited to the ProteomeXchange database (identifier code: PXD008904). Among the identified proteins, 5242 were detected in at least two of the three tested replicates (Table S2).

Proteome‐level comparisons between P178SCR and P178Mock and between Lx9801SCR and Lx9801Mock were used to define the proteins affected by the *P. polysora* infection in the resistant and susceptible genotypes. First, we determined the cut‐off for up‐ or down‐regulation based on the label‐specific experimental variation between three replicates for the four experimental groups (P178SCR, P178Mock, Lx9801SCR and Lx9801Mock). Then, the frequency distribution of the fold deviation from the mean of each group was calculated as described previously (Datta *et al*., 2010, 2017). The experimental variation was <1.2‐fold for around 98%, 93%, 96% and 98% of the ratios for P178SCR, P178Mock, Lx9801SCR and Lx9801Mock groups, respectively (Figure S1, Table S2). Based on these results, the cut‐off used for identification of the differentially accumulated proteins was set at 1.2‐fold. A total of 2126 proteins were differentially accumulated [i.e. fold‐change >1.2 (*P *<* *0.05)]. Among these differentially accumulated proteins, 1489 were identified in the resistant genotype (687 up‐regulated and 802 down‐regulated) (Table S3, Figure 1j), while 1035 were identified in the susceptible genotype (571 up‐regulated and 464 down‐regulated) (Table S4, Figure 1j).

### Functional analysis of differentially accumulated proteins

**Figure 2:**
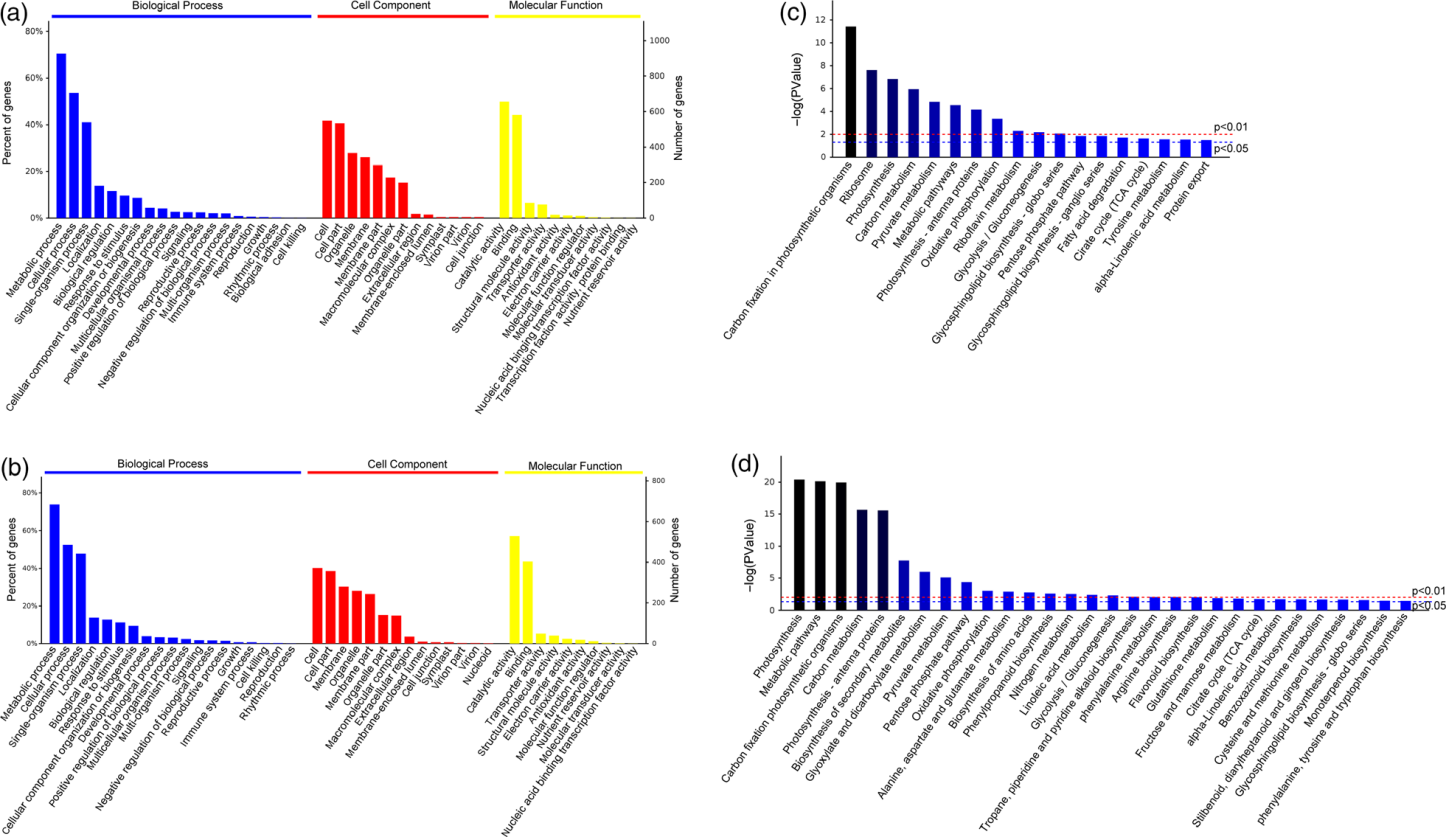
Functional annotation of differentially accumulated proteins. Gene Ontology (GO) enrichment analysis of the differentially accumulated proteins in the resistant (a) and susceptible (b) genotypes. Kyoto Encyclopedia of Genes and Genomes (KEGG) pathway enrichment analysis of the differentially accumulated proteins in resistant (c) and susceptible (d) genotypes.

According to the GO term enrichment analysis, the differentially accumulated proteins were classified into three main categories (i.e. cellular component, molecular function and biological process). The main biological processes GO terms for the resistant and susceptible genotypes were metabolic process, cellular process and single‐organism process (Figure 2a,b). The main cellular components GO terms were cell, cell part and organelle for the resistant genotype, and cell, cell part and membrane for the susceptible genotype (Figure 2a,b). The main molecular function GO terms were catalytic activity, binding and structural molecule activity for the resistant genotype, and catalytic activity, binding and transporter activity for the susceptible genotype (Figure 2a,b).

To characterize the differences in the metabolic pathways of the resistant and susceptible genotypes during the *P. polysora* infection, the differentially accumulated proteins underwent a KEGG pathway enrichment analysis. A total of 113 and 105 pathways were enriched for the differentially accumulated proteins in resistant and susceptible genotypes (Figure S2). Among these pathways, 18 and 11 were enriched at the *P *≤* *0.05 and 0.01 levels, respectively, in the resistant genotype (Figure 2c). Meanwhile, 30 and 20 pathways were enriched at the *P *≤* *0.05 and 0.01 levels, respectively, in the susceptible genotype (Figure 2d). Of these pathways enriched at *P *≤* *0.05 level, the main pathways in the resistant genotype were carbon fixation in photosynthetic organisms, ribosome and photosynthesis, while the main pathways in the susceptible genotype were photosynthesis, metabolic pathways and carbon fixation in photosynthetic organisms.

### Expression pattern and subcellular localization of ZmREM1.3

**Figure 3:**
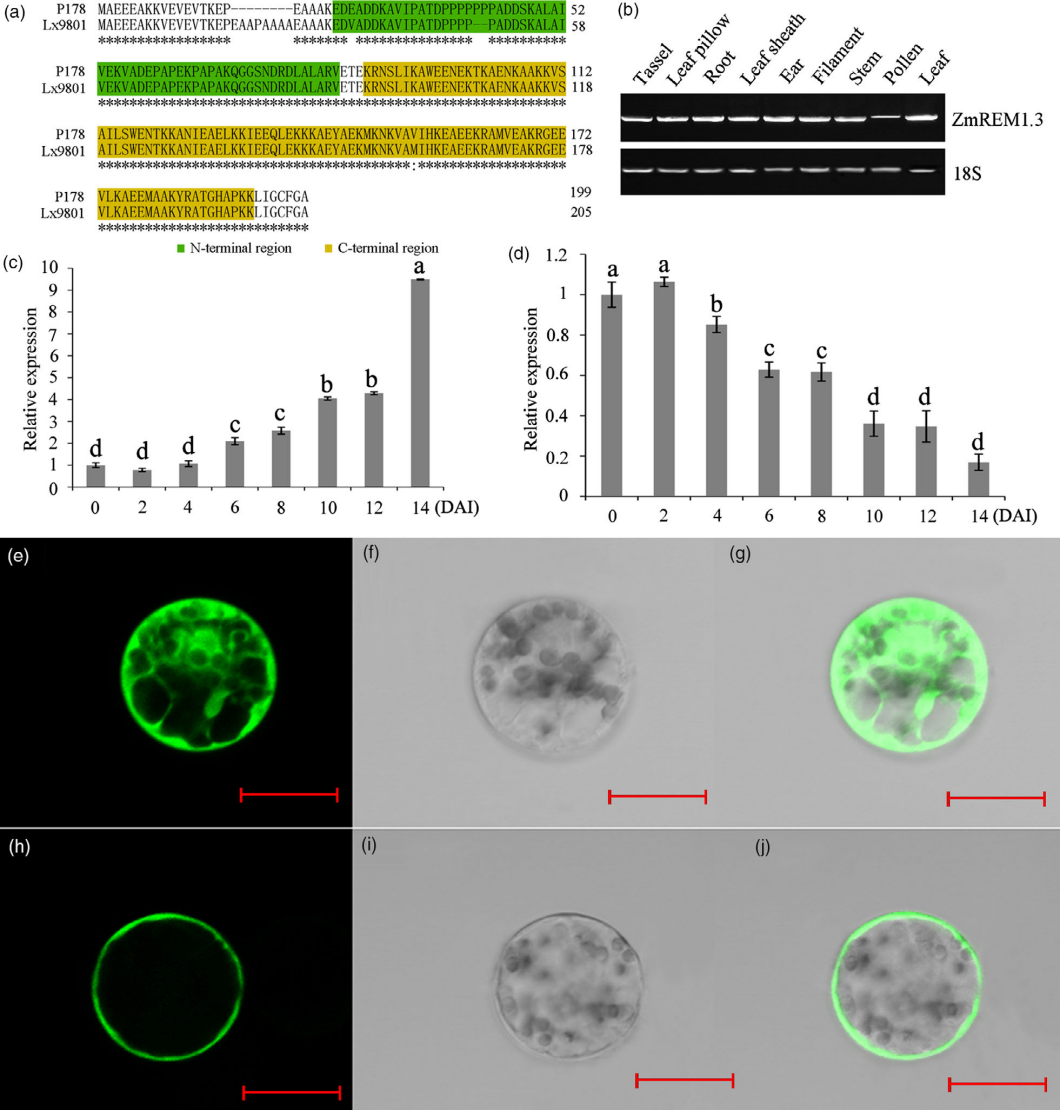
ZmREM1.3 structure, expression patterns and subcellular localization. (a) ZmREM1.3 structures and amino acid sequences in the resistant and susceptible genotypes. (b) *ZmREM1.3* transcript levels in different tissues according to semi‐quantitative RT‐PCR, with the 18S rRNA gene as a control. Relative expression of *ZmREM1.3* in the resistant line (P178) (c) and in the susceptible line (Lx9801) (d) at specific time points (days) after inoculation (DAI) as determined by qRT‐PCR. Transcript abundance was normalized against the 18S rRNA transcript level. Error bars represent standard errors from three independent experiments. A one‐way analysis of variance was completed, and statistically significant differences are indicated with lowercase letters (a, b, c and d) (*P *<* *0.05). Subcellular localization of CaMV35S:GFP (e–g) and CaMV35S:ZmREM1.3‐GFP (h–j) in maize protoplasts. (g, j) Merged fluorescence (e, h) and bright‐field (f, i) images. Scale bars = 20 μm.

An earlier study revealed that the remorin family member is important for biotic stress responses in tomato (Raffaele *et al*., 2009). Of all the differentially accumulated proteins, a defence‐related protein, remorin (B4G1B0), was selected for further analyses because the *P. polysora* infection significantly increased its abundance in the resistant genotype. An analysis using the Basic Local Alignment Search Tool (http://www.ncbi.nlm.nih.gov/BLAST/) revealed that the identified remorin protein contains both remorin‐C domain and remorin‐N domain (Figure 3a). Thus, it was named ZmREM1.3 according to the protein nomenclature described by Raffaele *et al*. (2007).

We examined the *ZmREM1.3* expression pattern in various maize tissues (i.e. tassel, leaf pillow, root, leaf sheath, ear, filament, stem, pollen and leaf tissues) by semi‐quantitative RT‐PCR. The *ZmREM1.3* gene appeared to be expressed in all analysed tissues, although at a relatively low level in pollen grains (Figure 3b). In combination with the expression data from qTeller (http://www.qteller.com/) (Figure S3), these results suggested that *ZmREM1.3* is constitutively and ubiquitously expressed in maize plants.

An investigation of the *ZmREM1.3* expression pattern in the resistant and susceptible genotypes in response to the *P. polysora* infection indicated that *ZmREM1.3* expression was up‐regulated in the resistant line, but down‐regulated in the susceptible genotype (Figure 3c,d). The *ZmREM1.3* expression patterns were consistent with the protein abundance changes in the resistant and susceptible genotypes infected with *P. polysora*. These observed differences between the resistant and susceptible genotypes implied that ZmREM1.3 is likely important for the host–pathogen interaction.

To determine the subcellular localization of ZmREM1.3, we created a vector that used the CaMV35S promoter to drive expression of ZmREM1.3‐GFP (CaMV35S‐ZmREM1.3‐GFP). Then, this vector was introduced into maize protoplasts. In contrast to the CaMV35S‐GFP fluorescence signals, which were detected throughout maize protoplast cells, the CaMV35S‐ZmREM1.3‐GFP signals were restricted to cell membranes (Figure 3e–j). These results showed that ZmREM1.3 appears to be membrane localized which does support previous findings of other remorins (Raffaele *et al*., 2013; Reymond *et al*., 1996).

### Phenotypic and physiological changes in *P**. **polysora‐*infected maize plants overexpressing *ZmREM1.3*

**Figure 4:**
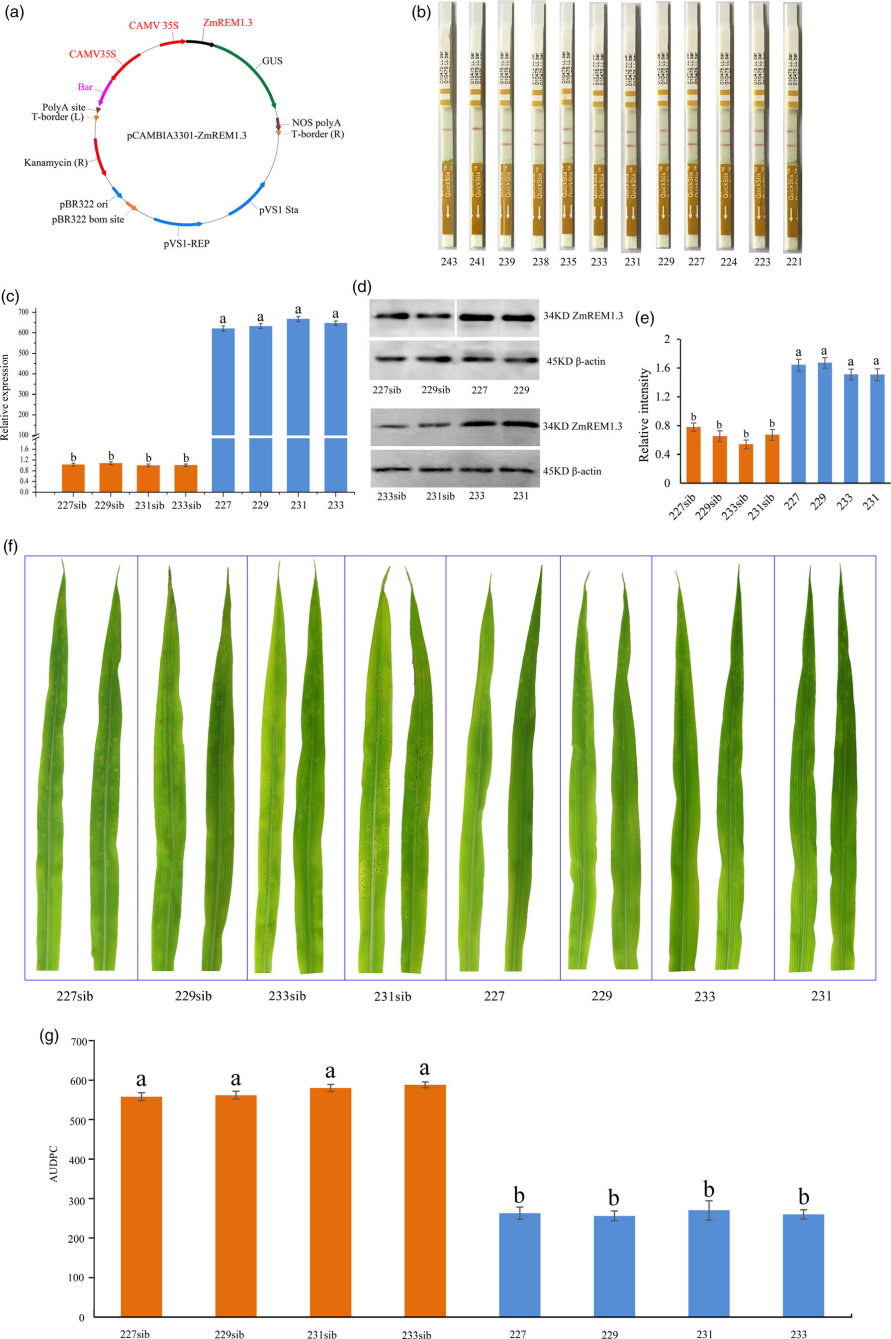
Resistance of maize plants overexpressing ZmREM1.3 to southern corn rust. (a) Map of the pCAMBIA3301‐ZmREM1.3 recombinant expression vector. (b) Analysis of *T*_1_ transformants with the QuickStix Kit for LibertyLink indicated 10 of 12 independent transformation events resulting in transgenic plants carrying *ZmREM1.3*. (c) *ZmREM1.3* expression levels in transgenic maize lines 227, 229, 231 and 233 as well as the corresponding non‐transgenic sib lines. The *ZmREM1.3* transcript level was normalized against the 18S rRNA transcript level. (d) Western blot analysis of ZmREM1.3 in transgenic maize lines 227, 229, 231 and 233 as well as the corresponding non‐transgenic sib lines, with β‐actin as the loading control. (e) The intensities of bands in the Western blot were measured by ImageJ, and the abundance was normalized against the β‐actin. (f) Macroscopic evaluation of southern corn rust symptoms on the leaves of transgenic maize lines 227, 229, 231 and 233 as well as the corresponding non‐transgenic sib lines at 14 days after inoculation. (g) Area under the disease progress curve (AUDPC) calculated daily between 8 and 14 days after inoculation for transgenic maize lines 227, 229, 231 and 233 as well as the corresponding non‐transgenic sib lines. Error bars represent the standard errors from three independent experiments. A one‐way analysis of variance was completed, and statistically significant differences are indicated with lowercase letters (a and b) (*P *<* *0.05).

To further functionally characterize ZmREM1.3, the *ZmREM1.3* coding sequence in P178 plants was placed under the control of the CaMV35S promoter (Figure 4a). The primary phosphinothricin‐resistant transgenic plants from independent transformation events (i.e. derived from different immature embryos) were tested for the presence of *ZmREM1.3* transgene. Of 12 independent transformation events, 10 resulted in transgenic plants carrying *ZmREM1.3* (Figures 4b, S4). These *T*_1_ plants were grown to maturity and self‐pollinated to produce both non‐segregating transgenic positive and negative (non‐transgenic sib lines) lines (*T*_3_ generation). Four *T*_3_ non‐segregating lines (227, 229, 231 and 233 as well as non‐transgenic sib lines 227sib, 229sib, 231sib and 233sib) were used for subsequent analyses. The *ZmREM1.3* expression levels were higher in the transgenic lines than in the corresponding non‐transgenic sib lines (Figure 4c). Moreover, a Western blot analysis using rabbit polyclonal antibody of ZmREM1.3 confirmed that ZmREM1.3 was more abundant in the four *ZmREM1.3*‐overexpressing lines than in the corresponding non‐transgenic sib lines (Figure 4d,e).

**Figure 5:**
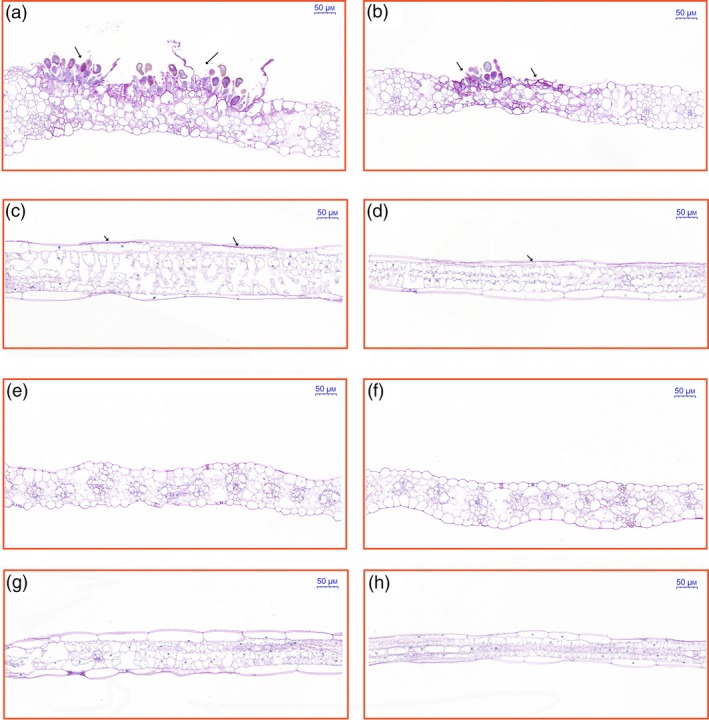
Cellular responses to the *P. polysora* infection in transgenic maize plants. Transgenic maize plants inoculated with *P. polysora* spores were grown until 14 days after inoculation. Leaf cross sections for lines 231sib (a), 233sib (b), 231 (e) and 233 (f) as well as leaf longitudinal sections for lines 231sib (c), 233sib (d), 231 (g) and 233 (h) were stained with periodic acid‐Schiff reagents. The distribution of fungal hyphae was observed using the Pannoramic MIDI scanner. Positions indicated by arrows represent the infection sites. Scale bars = 50 μm. All results shown are representative of three independent experiments.

To examine the responses of transgenic plants to *P. polysora* infection, transgenic and non‐transgenic sib lines were grown in a greenhouse. Specifically, fewer light‐orange pustules were detected in the transgenic lines at 8 days after inoculation, and dust‐like, gold spores were exposed at 9 days after inoculation. Meanwhile, for the non‐transgenic sib lines, the pustules were observed at 7 days after inoculation and spores were exposed at 8 days after inoculation. Additionally, spores were more abundant and larger on the non‐transgenic sib lines than on the corresponding transgenic lines. Furthermore, the spores on the non‐transgenic sib lines were present on the entire leaf, unlike the spores on the transgenic lines, which were present only at the leaf tip (Figure 4f). Lines 227, 229, 231 and 233 were considerably more resistant to *P. polysora* than the corresponding non‐transgenic sib lines. The area under the disease progress curve (AUDPC) value has been used as an indicator of the slow‐rusting characteristic of plants (Bailey *et al*., 1987; Hurni *et al*., 2015; Ji, 2006). In this study, disease severity was quantitatively assessed based on percentage of plants exhibiting symptoms daily between 8 and 14 days after inoculation. We observed a significant difference in the AUDPC values between the transgenic and the corresponding non‐transgenic sib lines, revealing the delayed disease symptom development in the four transgenic lines (Figure 4g). These results implied that overexpressing *ZmREM1.3* in maize improves resistance to *P. polysora*. To further clarify the morphological changes at the cellular level, longitudinal and cross sections of *P. polysora*‐infected leaves from two transgenic (231 and 233) and the corresponding non‐transgenic sib lines (231sib and 233sib) were examined. In the non‐transgenic sib lines, *P. polysora* hyphae were observed inside the leaf tissue, while the epidermal and palisade cells were destroyed and fungal spores were detected on the leaf surface (Figure 5a–d). In the transgenic lines, relatively few hyphae were observed in the leaf tissue and the leaf cell shapes were normal (Figure 5e–h).

We also examined the phenotype of A188 and B73 in response to *P. polysora* infection, since the ZmREM1.3‐overexpressing transgenic plants were developed in Hi‐II, which is an *F*_1_ hybrid from these two inbred lines. At 14 days after inoculation, dust‐like, gold spores appeared on the surface of both A188 and B73 leaves (Figure S5a), and AUDPC values also showed that these two inbred lines were susceptible to *P. polysora* infection (Figure S5b). In addition, we analysed the expression levels of *ZmREM1.3* in both A188 and B73. After *P. polysora* infection, the transcript levels of *ZmREM1.3* decreased in both inbred lines (Figure S5c,d). Furthermore, we examined the protein levels after *P. polysora* and mock infection using Western blot analysis (Figure S5e,f). Our results showed that the ZmREM1.3 protein abundance was also down‐regulated after *P. polysora* infection. These results suggested that the resistance of the four transgenic lines was due to the overexpression of *ZmREM1.3*.

### ZmREM1.3 affects the accumulation of plant hormones and the expression of defence genes

**Figure 6:**
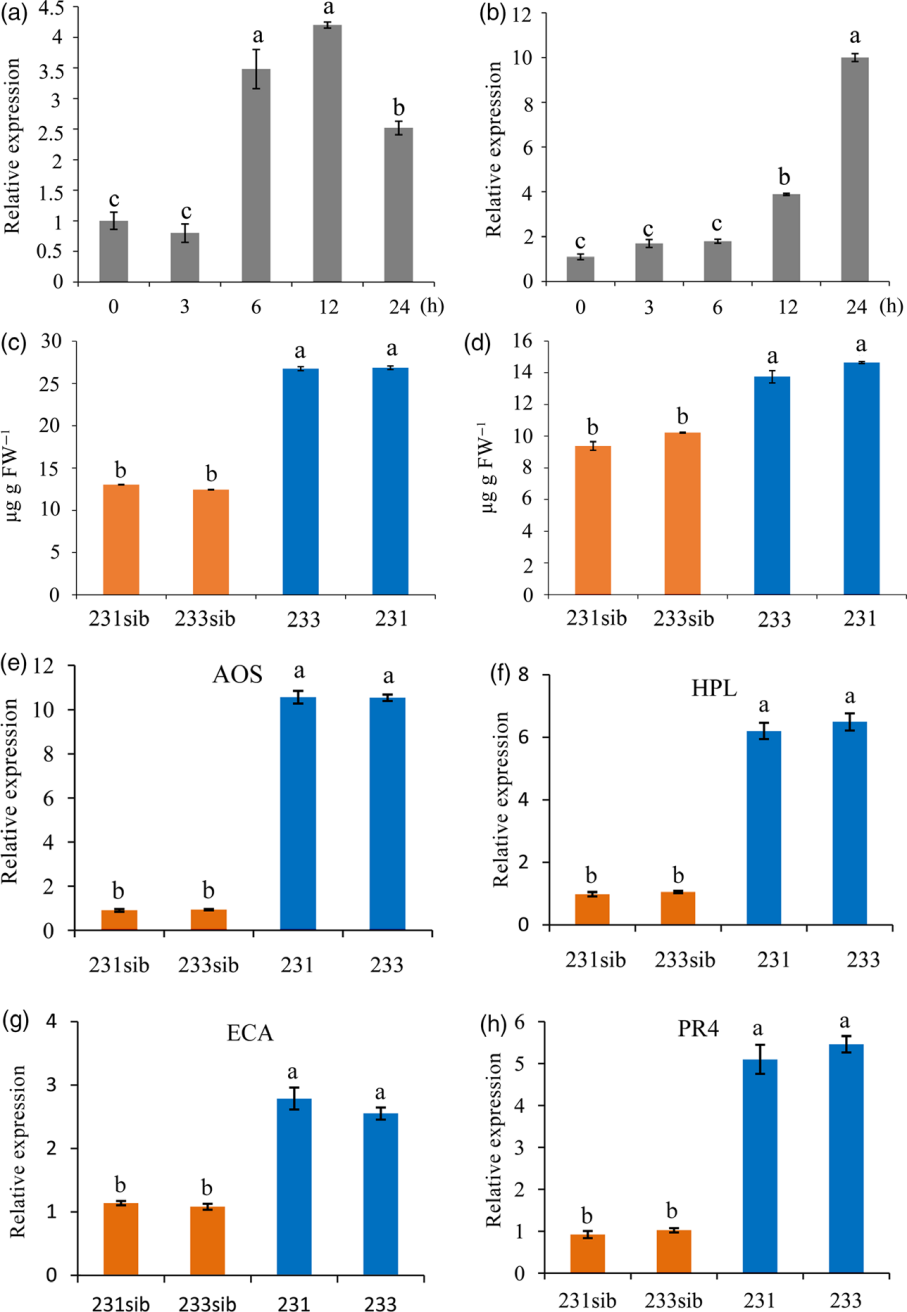
Role of plant hormone and defence signalling in the resistance conferred by ZmREM1.3. Seedlings of resistant line P178 were treated with salicylic acid (SA) and jasmonic acid (JA). The *ZmREM1.3* expression pattern was examined by qRT‐PCR after 0, 3, 6, 12 and 24 h. Transcript abundance was normalized against the 18S rRNA transcript level. Transgenic maize plants inoculated with *P. polysora* spores were grown until 14 days after the inoculation. Endogenous hormone contents were examined by HPLC‐MS/MS. Both SA (a) and JA (b) up‐regulated the *ZmREM1.3* expression level. Endogenous SA (c) and JA (d) levels in transgenic maize lines 231 and 233 as well as the corresponding non‐transgenic sib lines. The expression levels of defence‐related genes *AOS* (e), *HPL* (f), *ECA* (g) and *PR4* (h) in transgenic maize lines 231 and 233 as well as the non‐transgenic sib lines were examined by qRT‐PCR. Error bars represent the standard errors from three independent experiments. A one‐way analysis of variance was completed, and significant differences are indicated with lowercase letters (a, b, c and d) (*P *<* *0.05).

Plant hormones are central regulators of plant defence, and the intricate network of plant hormone signalling pathways enables plants to activate appropriate and effective defence responses against pathogens (Berens *et al*., 2017). To study potential relationships between ZmREM1.3 and plant hormone signalling pathways, we examined the *ZmREM1.3* expression levels in response to SA and JA treatments. As shown in Figure 6a,b, the *ZmREM1.3* expression levels were significantly up‐regulated by SA and JA. We also quantified the SA and JA concentrations to assess whether the resistance conferred by *ZmREM1.3* overexpression was correlated with the accumulation of plant hormones in two *T*_3_ non‐segregating lines (231 and 233 as well as non‐transgenic sib lines 231sib and 233sib). The SA and JA contents were higher in the two transgenic lines than in the two corresponding non‐transgenic sib lines following the *P. polysora* infection (Figure 6c,d).

We also examined the expression levels of four well‐characterized maize defence marker genes. Previous investigations demonstrated that allene oxide synthase (*AOS*) and hydroperoxide lyase (*HPL*) are involved in defence responses to pathogens (Conrath *et al*., 2006; Feussner and Wasternack, 2002; Mei *et al*., 2006). After *P. polysora* infection, *AOS* and *HPL* transcript levels were significantly higher in the *ZmREM1.3*‐overexpressing lines than in the corresponding non‐transgenic sib lines (Figure 6e,f). Meanwhile, endochitinase A (*ECA*) and pathogenesis‐related 4 (*PR4*) were identified as pathogen‐inducible defence genes in previous studies (Doehlemann *et al*., 2008; Huffaker *et al*., 2011; Matsui, 2006). The transcript levels for these two defence genes were also significantly higher in the *ZmREM1.3*‐overexpressing lines than in the corresponding non‐transgenic sib lines (Figure 6g,h).

### Mutant analysis confirmed the positive role of ZmREM1.3 in SCR resistance

**Figure 7:**
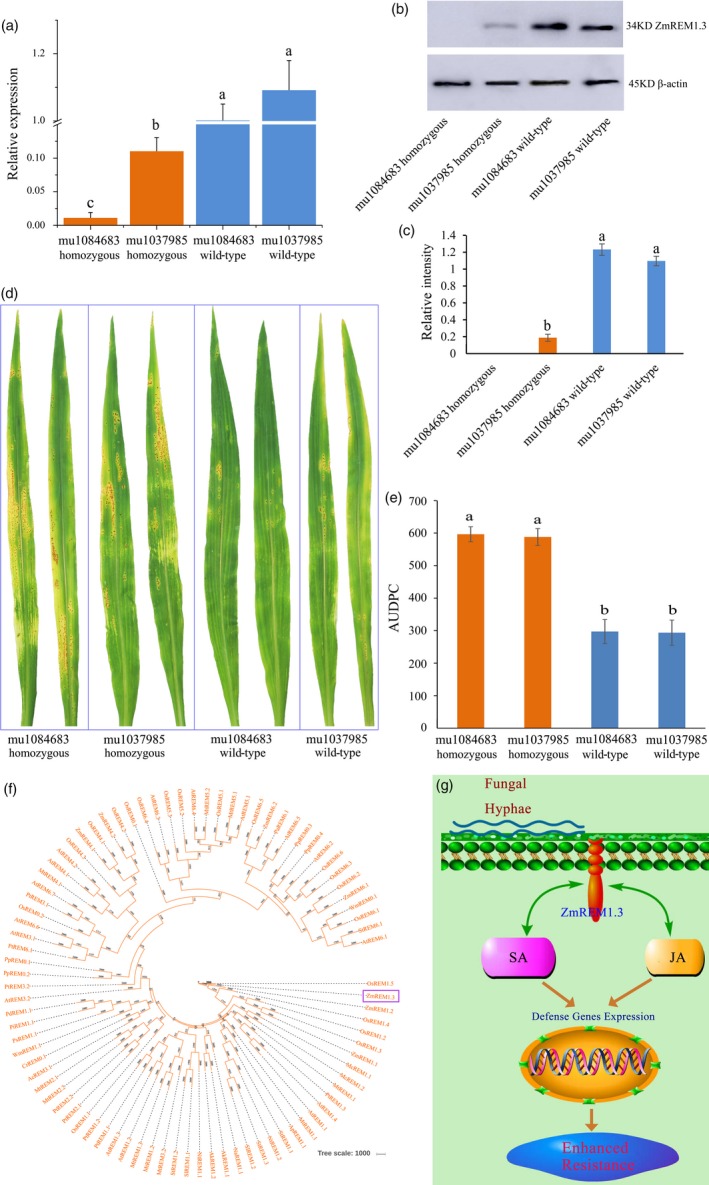
Mutant analysis confirmed the positive role of ZmREM1.3 in southern corn rust resistance and a proposed model of the resistance mechanism. (a) *ZmREM1.3* expression levels in two UniformMu mutant lines (mu1084683 and mu1037985) as well as the corresponding wild‐type lines. The *ZmREM1.3* transcript level was normalized against the 18S rRNA transcript level. (b) Western blot analysis of ZmREM1.3 in two UniformMu mutant lines (mu1084683 and mu1037985) as well as the corresponding wild‐type lines, with β‐actin as the loading control. (c) The intensities of bands in the Western blot were measured by ImageJ, and the abundance was normalized against the β‐actin. (d) Macroscopic evaluation of southern corn rust symptoms on the leaves of two homozygous mutant lines (mu1084683 and mu1037985) as well as the corresponding wild‐type lines at 14 days after inoculation. (e) Area under the disease progress curve (AUDPC) calculated daily between 8 and 14 days after inoculation for two homozygous mutant lines (mu1084683 and mu1037985) as well as the corresponding wild‐type lines. Error bars represent the standard errors from three independent experiments. A one‐way analysis of variance was completed, and statistically significant differences are indicated with lowercase letters (a, b and c) (*P *<* *0.05). (f) Phylogenetic analysis of remorin family proteins. The identified ZmREM1.3 and previously classified remorin family proteins were analysed using PHYLIP. ZmREM1.3 clustered in Group 1. (g) Proposed model of the mechanisms underlying the ZmREM1.3‐mediated resistance to *P. polysora*. In response to a of *P. polysora* infection, transgenic maize plants constitutively expressing *ZmREM1.3* accumulate salicylic acid (SA) and jasmonic acid (JA), resulting in up‐regulated the expression of defence‐related genes involved in the resistance to the biotroph *P. polysora*.

To confirm the positive role of ZmREM1.3 in SCR resistance, two UniformMu mutants with different insertions in *ZmREM1.3* (GRMZM2G122937) were identified (Figure S6), one of which carries a transposon insertion (mu1084683 in UFMu‐10378) in the *ZmREM1.3* coding region, and the other has a transposon insertion (mu1037985 in UFMu‐03259) in 64 bp upstream of the *ZmREM1.3* coding region. qPCR analysis revealed that the *ZmREM1.3* expression levels were significantly lower in the homozygous mutant lines than in the corresponding wild‐type lines (Figure 7a). Moreover, Western blot analysis showed that there is no ZmREM1.3 protein accumulation in the mu1084683 insertion homozygous line, and the protein abundance is lower in the mu1037985 insertion homozygous lines than in the corresponding wild‐type lines (Figure 7b,c).

We further examined the phenotypes of the homozygous mutant lines and corresponding wild‐type lines in response to *P. polysora* infection. Spores were more abundant on the homozygous mutant lines than that on the corresponding wild‐type lines (Figure 7d,e). The results revealed that the homozygous mutant lines were significantly more susceptible than the corresponding wild‐type lines. These results further confirmed the positive role of ZmREM1.3 in maize resistance to SCR.

## Discussion

Plant diseases caused by pathogens, such as viruses, bacteria and fungi, are responsible for major economic losses to agriculturally important crops worldwide (Kangasjarvi *et al*., 2012). Rust pathogens are economically significant and difficult to manage because of their dynamic population structure, rapid evolution and wide dispersal of anemochorous spores (Gill *et al*., 2018). *P. polysora* is a biotrophic fungal pathogen that causes SCR (Raid *et al*., 1988). Previous genetic studies revealed several SCR‐resistance loci (Chen *et al*., 2004; Wu *et al*., 2015b; Zhou *et al*., 2007), but SCR‐resistance genes have not been identified. In this study, SCR‐resistant (P178) and SCR‐susceptible (Lx9801) maize inbred lines underwent a proteome‐level investigation to elucidate the complex interaction between *P. polysora* and maize. Our results demonstrate that overexpression of the remorin protein (ZmREM1.3) in maize confers SCR resistance likely *via* a SA/JA‐mediated defence pathway.

### Changes to photosynthesis and energy metabolism induced by *P**. **polysora*

The allocation of resources for plant immune responses and the biosynthesis of protective compounds in infected tissues require energy (Kangasjarvi *et al*., 2012). However, several studies have reported that photosynthetic activities are down‐regulated in response to diverse pathogens, with plants shifting towards non‐assimilatory metabolic activities (Bolton, 2009; Kerchev *et al*., 2012; Major *et al*., 2010; Roberts and Paul, 2006). Such metabolic shifts are plant‐driven and tightly coordinated. In the current study, we observed that the abundance of the photosystem I and II subunits and the chlorophyll a/b‐binding antenna complex decreased in the susceptible genotype after the *P. polysora* infection (Table S4). Decreased photosynthetic activities in pathogen‐infected tissue were proposed as part of a plant strategy to shift from photosynthetic and other assimilatory metabolic activities to respiration (Major *et al*., 2010). The resulting demand for carbohydrates and energy would be satisfied through increased activities of cell wall invertases, hexose transporters, the oxidative pentose phosphate pathway and respiratory metabolism. Such reprogramming of primary carbon metabolism may further enhance the expression of defence‐related genes and stimulate the production of secondary compounds with antimicrobial activities (Bolton, 2009). However, the observed high turnover of photosynthetic proteins including photosystem I and II subunits and the chlorophyll a/b‐binding proteins in the resistant genotype suggests that SCR‐resistant maize plants may induce defence mechanisms that protect the photosynthetic machinery to maintain photosynthetic efficiency in response to a *P. polysora* infection.

### Changes to protein and amino acid metabolism due to *P**. **polysora*

Ribosomal proteins are highly conserved components of basal cellular organelles, and they are primarily involved in protein synthesis (Liu *et al*., 2014). However, ribosomal proteins are suspected to have additional functions related to disease or stress resistance (Nagaraj *et al*., 2016; Warner and Mcintosh, 2009). In the present study, we revealed that the abundance of many ribosomal proteins was affected by *P. polysora* infection, with 21 down‐accumulated and 54 up‐accumulated proteins in the resistant genotype (Table S3) and 10 down‐regulated and 16 up‐regulated proteins in the susceptible genotype (Table S4). The differences in the accumulation of these proteins between the resistant and susceptible genotypes following *P. polysora* infection imply these ribosomal proteins may be involved in the maize–*P. polysora* interaction.

Many plant stress defence compounds are derived from amino acid precursors. Plant defences against pathogens include the biosynthesis of compounds exhibiting antimicrobial or toxic activities. These compounds are either constitutively produced in developing plants or are synthesized in an inducible manner in response to biotic stresses (Ahuja *et al*., 2012; Bednarek and Osbourn, 2009). Our KEGG pathway enrichment analysis revealed that the susceptible genotype was enriched with differentially abundant proteins related to aspartate and glutamate metabolism (Figure 2d). Aspartate can be metabolized to asparagine by asparagine synthetase, while glutamate can be converted to glutamine by glutamine synthetase. Changes to several steps of the aspartate biosynthetic pathway can have profound effects on plant–microbe interactions (Jander and Joshi, 2010). Altered glutamate metabolism in plant hosts due to different pathogens appears to either support the ongoing defence response or is exploited by the pathogen to promote and facilitate infection (Seifi *et al*., 2013). In this study, the observed enhanced synthesis of asparagine and glutamine in the infected tissues of the susceptible genotype may have decreased the production of defence compounds, potentially leading to the reassimilation of ammonium and remobilization of nitrogen, which enhanced *P. polysora* infection of maize.

### Maize plants overexpressing *ZmREM1.3* are resistant to southern corn rust

Remorins, which are plant‐specific proteins encoded multigene family, are present in all land plants, including ferns and mosses (Jarsch and Ott, 2011). The first described remorin was pp34, which is phosphorylated potato protein with a molecular mass of 34 kDa (Jacinto *et al*., 1993). This protein was renamed as a remorin because of its hydrophilic profile and its ability to attach to the plasma membrane (PM) (Raffaele *et al*., 2013; Reymond *et al*., 1996). In this study, we observed that CaMV35S‐ZmREM1.3‐GFP was localized to membrane domains, possibly reflecting an enrichment of this protein in lipid rafts (Figure 3e–j). Several proteomic examinations of the PM of tobacco leaves and *A. thaliana* seedlings revealed the presence of remorins (Bhat and Panstruga, 2005; Laloi *et al*., 2007; Lefebvre *et al*., 2007; Marmagne *et al*., 2004; Mongrand *et al*., 2004; Nelson *et al*., 2006; Sazuka *et al*., 2004; Valot *et al*., 2006; Watson *et al*., 2003), with the C‐terminal anchor being indispensable and sufficient for the binding of remorins to the PM. Another study indicated that the localization of the remorin *StREM1.3* to the PM is required for restricting potato virus X movement (Perraki *et al*., 2012). The predicted coiled‐coil domains within the C‐terminal domain are believed to be common among all remorin proteins (Raffaele *et al*., 2007). However, significant differences among remorins, especially in the N‐terminal regions, resulted in the remorin family being subdivided into the following six groups (Figure 7f): Group 1 (Subgroups 1a and 1b: canonical remorins with a proline‐rich N‐terminal region); Group 2 (remorins in legumes and poplar); Group 3 (short remorins); Group 4 (remorins with alternative proline‐rich N‐terminal regions); Group 5 (remorins with low N‐terminal proline content); and Group 6 (long remorins) (Raffaele *et al*., 2007). Our phylogenetic analysis suggested that ZmREM1.3, which contains remorin‐C and remorin‐N domains, belongs to Subgroup 1b (Figures 7f and S7).

Previous studies have revealed the important functions of remorins during plant responses to abiotic and biotic stresses. For example, *A. thaliana* plants overexpressing *MiREM* and *SiREM6* are tolerant to dehydration and salinity stresses (Checker and Khurana, 2013; Yue *et al*., 2014). Additionally, a genetic analysis indicated that *ZmREM6.3* confers quantitative resistance against northern leaf blight (Jamann *et al*., 2016). In contrast, nodule formation is enhanced by the expression of remorin‐encoding genes, including *LjSYMREM1* in *Lotus japonicus* (Toth *et al*., 2012), *MtSYMREM1* in *Medicago truncatula* (Lefebvre and Dangl, 2010), and *GmREM2.1* in soybean (Son *et al*., 2015). Moreover, *NbREM1.3* and *StREM1.3* being susceptible genes against oomycete *P. infestans* has been demonstrated (Bozkurt *et al*., 2014). However, little is known about the role of *ZmREM1.3* in response to biotroph *P. polydora*. In this study, we observed that the *ZmREM1.3* transcript level and the abundance of the encoded protein increased in the resistant genotype, but decreased in the susceptible genotype, in response to the *P. polysora* infection. Thus, ZmREM1.3 may influence the host–pathogen interaction. Experiments involving transgenic plants and UniformMu mutant plants provided further evidence that ZmREM1.3 enhances SCR resistance in maize (Figures 4c–g and 7a–e).

### Potential defence pathways involved in ZmREM1.3‐mediated southern corn rust resistance

Similar to a previous study that concluded the expression of remorin family genes can be induced by plant hormones (Yue *et al*., 2014), we observed that exogenously applied JA and SA up‐regulated *ZmREM1.3* expression in maize (Figure 6a,b). Therefore, the SCR resistance conferred by overexpression of *ZmREM1.3* may be associated with defence responses regulated by hormone signalling pathways. Stress resistance triggered by defence‐related hormones such as SA and JA has been described in maize and other plant species (Djonovic *et al*., 2007; Morris *et al*., 1998). Salicylic acid is a positive regulator of immunity against biotrophs and hemibiotrophs with a biotrophic phase during the early stages of infection. Meanwhile, JA is a positive regulator of immunity against necrotrophs (Glazebrook, 2005). Our analyses of SA and JA contents in transgenic and the non‐transgenic sib lines indicated that SA and JA levels were much higher in *ZmREM1.3*‐overexpressing lines than in the non‐transgenic sib lines (Figure 6c,d). These results suggested that SA and JA pathways are activated in defence responses against the biotrophic *P. polysora*. Our results also indicated that despite a well‐documented reciprocal inhibition, the relationship between SA and JA is not always antagonistic. This is consistent with recent reports that JA can positively regulate plant resistance to biotrophic pathogens (Guerreiro *et al*., 2016; Liu *et al*., 2016; Tamaoki *et al*., 2013; Yamada *et al*., 2012).

In addition to mediating the production of JA and SA, ZmREM1.3 also promoted increased transcript abundance for genes encoding defence signalling proteins. *AOS* and *HPL* are well‐known genes in JA‐regulated defence pathways. Previous studies have shown that overexpression of the *AOS* gene in rice increased endogenous JA levels, followed by enhanced resistance to fungal infection (Mei *et al*., 2006). The *T. asperellum*‐mediated systemic disease protection of cucumber plants from a bacterial leaf pathogen was associated with increased levels of *HPL* transcripts (Yedidia *et al*., 2003), and overexpression of the *HPL* gene in *Arabidopsis* resulted in enhanced resistance against *B. cinereal* (Shiojiri *et al*., 2006). *PR4* and *ECA* have been shown to be pathogen inducible in microarray experiments (Doehlemann *et al*., 2008). *ECA* encode chitinase proteins likely to have direct antifungal activity through degradation of fungal cell walls. The up‐regulated expression of these genes in *ZmREM1.3*‐overexpressing lines suggested that basal defence responses are enhanced in *ZmREM1.3*‐overexpressing lines, ultimately resulting in *P. polysora* resistance.

Our comparative proteomics analysis combined with genetic, biochemical and physiological investigations suggests ZmREM1.3 along with SA/JA‐mediated defence pathways regulates the SCR resistance of maize plants. We developed a model of the potential resistance mechanisms (Figure 7g). In response to *P. polysora* infection, transgenic maize plants constitutively expressing *ZmREM1.3* significantly increase SA and JA production, forming a positive feedback loop affecting *ZmREM1.3* expression. Synergistic interactions between the SA and JA pathways induce the expression of antimicrobial or defence‐related genes mediating resistance against the biotrophic pathogens *P. polysora*.

## Experimental procedures

### Plant materials and *Puccinia polysora* inoculations

To investigate the effects of *P. polysora* infections on maize, inbred lines P178 (SCR‐resistant) and Lx9801 (SCR‐susceptible) underwent a phenotype examination and an iTRAQ analysis. Plants were grown in a greenhouse under a 15‐h light (28 °C)/9‐h (25 °C) photoperiod to 4–5 leaf stage. Then, those plants were inoculated with a solution consisting of 0.02% Tween‐20 (v/v) and approximately 10^5^–10^6^ *P. polysora* spores per mL. *P. polysora* spores (race PP.9) were collected from *P. polysora*‐infected susceptible adult Lx9801 plants exhibiting SCR symptoms. Plants were inoculated using an air‐assisted sprayer, with 5 mL spores solution per plant, and then incubated for 24 h at 25 °C under high humidity (approximately 95%) in darkness. The plants were then incubated under a 15‐h light (28 °C)/9‐h (25 °C) photoperiod with 80% humidity. Control plants were mock‐inoculated with inoculation buffer. Leaves from five fresh plants were harvested at 0, 2, 4, 6, 8, 10, 12 and 14 days after inoculation. Three replicates were collected at each time point. The collected leaves were quickly frozen in liquid nitrogen and stored at −80 °C until analysed.

### Protein preparation

Maize leaves of four groups (P178mock, P178SCR, Lx9801mock and Lx9801SCR) at 14 days after inoculation were used for proteomic analysis. Ten leaves from five fresh plants for each group were collected. *P. polysora* inoculation (P178SCR and Lx9801SCR) and mock‐inoculation control (P178mock and Lx9801mock) groups were analysed in one 4‐plex iTRAQ set. Three independent biological replicates were applied (Figure 1i). Approximately 3 g frozen leaves of each group were ground to a powder in liquid nitrogen. The ground material was treated with a 10% (w/v) trichloroacetic acid/acetone solution containing 65 mm dithiothreitol at −20 °C for 1 h. The solution was centrifuged at 10 000 ***g*** for 45 min, after which the supernatant was discarded and the precipitate was vacuum‐dried and solubilized in SDT buffer (4% SDS, 100 mm dithiothreitol and 150 mm Tris‐HCl, pH 8.0) with 1/10 the original volume. Samples were boiled for 3 min ultrasonicated (10 times at 80 W with 10 s pulses and 15 s intervals), and incubated at 100 °C for 3 min. The crude extract was centrifuged at 13 000 ***g*** for 10 min at 25 °C. The protein concentration of the collected supernatants was determined using the BCA Protein Assay Reagent (Promega, Madison, WI). The supernatants were stored at −80 °C until analysed.

### Protein digestion and iTRAQ labelling

Proteins were digested using trypsin (Promega) according to the FASP procedure described by Wisniewski *et al*. (2009) and Wu *et al*. (2016). The resulting peptides were labelled using the 4‐plex and 8‐plex iTRAQ reagents according to the manufacturer's instructions (Applied Biosystems). Each iTRAQ reagent was dissolved in 70 μL ethanol and added to separate peptide mixtures. The samples were labelled as (9801SCR)‐114, (9801Mock)‐115, (P178SCR)‐116 and (P178Mock)‐117, and were multiplexed and vacuum‐dried.

### Peptide fractionation by strong cation exchange chromatography

The iTRAQ‐labelled peptides were fractionated by strong cation exchange chromatography using the AKTA Purifier system (GE Healthcare) as previously described (Wu *et al*., 2016). All samples were stored at −80 °C until analysed by liquid chromatography–tandem mass spectrometry (LC‐MS/MS).

### Liquid chromatography–electrospray ionization tandem mass spectrometry

A Q Exactive mass spectrometer coupled to Easy‐nLC (Thermo Fisher Scientific) was used to conduct LC‐MS/MS analyses as previously described (Wu *et al*., 2016). The mass spectrometry proteomics data have been deposited to the ProteomeXchange Consortium via the PRIDE (Vizcaino *et al*., 2016) partner repository with the dataset identifier PXD008904 (Username: reviewer28166@ebi.ac.uk, Password: FREgEW3k).

### Sequence database searches and data analysis

Raw data files were converted to MGF files with Proteome Discoverer 1.4 (Thermo Fisher Scientific). The default value (100%) was used for the allowed co‐isolation interference. The decoy and UniProt Plant databases (Uniprot Zeamays) (http://www.uniprot.org) containing 132 441 sequences (downloaded on May 8, 2016) were searched using the resulting files and the Mascot search engine (version 2.2; Matrix Science, London, UK). The search parameters were as follows: peptide mass tolerance: ±20 ppm; MS/MS tolerance: 0.1 Da, maximum missed cleavages: 2; potential variable modifications: oxidation (M) and iTRAQ 4‐plex (Y); and fixed modifications: carbamidomethyl (C), iTRAQ 4‐plex (N‐term) and iTRAQ 4‐plex (K). The integration window tolerance was set as 20 ppm. The peptide charge state was set as +2 to +3. The cut‐off for the global false discovery rate (FDR) for identifying peptides and proteins was set as 0.01. The quantitative protein ratios were weighted and normalized based on the median ratio using the Mascot program. Student's *t*‐tests were completed to determine the significance of any differences between samples. Differentially accumulated proteins were those exhibiting an abundance fold‐change of >1.2 (*P *<* *0.05) in at least two replicates. Additionally, the presence/absence of proteins was based on whether proteins were detected in only one material in at least two replicates. The biological coefficient of variation (CV) of each protein was also calculated.

The Blast2GO program (https://www.blast2go.com/) was used to functionally annotate differentially accumulated proteins with Gene Ontology (GO) terms in the three main categories (i.e. cell component, biological process and molecular function). The differentially accumulated proteins were also annotated with terms in the Kyoto Encyclopedia of Genes and Genomes (KEGG) database using the KAAS program (http://www.genome.jp/kaas-bin/kaas_main) to identify enriched pathways.

### Plant hormone treatment and sampling

The P178 plants were treated with salicylic acid (SA) or jasmonic acid (JA) as previously described (Wu *et al*., 2014). Leaves from five fresh plants were collected at 0, 3, 6, 12 and 24 h after the hormone treatments. Three replicates were collected at each time point. The collected leaves were quickly frozen in liquid nitrogen and stored at −80 °C until analysed.

### Gene expression analysis by a quantitative real‐time polymerase chain reaction

A quantitative real‐time polymerase chain reaction (qRT‐PCR) assay was conducted to investigate the expression‐level changes of *ZmREM1.3* and four defence‐related genes. Total RNA was extracted from the collected leaves using the Total RNA kit (Takara, Dalian, China). The Two‐Step Prime Script™ RT Reagent Kit with gDNA Eraser (Takara) was used to synthesize cDNA. Three biological replicates were prepared for each sample. The qRT‐PCR was completed using the LightCycler® 480II Real‐Time PCR Detection System (Roche, Switzerland). The 18S rRNA gene served as the endogenous control. Relative gene expression level was quantified according to the 2^−ΔΔCt^ method (Livak and Schmittgen, 2001).

### Semi‐quantitative real‐time polymerase chain reaction

We collected specific tissues (i.e. tassel, leaf pillow, root, leaf sheath, ear, filament, stem, pollen and leaf tissues) from P178 plants for a subsequent analysis of *ZmREM1.3* expression patterns. Three replicates were collected for each tissue, with each replicate consisting of samples derived from five fresh plants. Total RNA was extracted from each tissue using the Total RNA kit (Takara, Dalian, China).

First‐strand of cDNA was synthesized using the Prime Script™ RT Reagent Kit with gDNA Eraser (Takara, Dalian, China), and Oligo dT Primer was used. The 18S rRNA gene was used as the endogenous reference.

### Subcellular localization

The *ZmREM1.3* coding sequence in P178 plants was amplified using gene‐specific primers (Table S1) and cloned into the pCAMBIA1304 binary vector so that its expression was under the control of the cauliflower mosaic virus (CaMV) 35S promoter. The CaMV35S‐ZmREM1.3‐GFP and CaMV35S‐GFP constructs were firstly purified using plasmid purification kit (TaKaRa, Dalian, China), then transiently expressed in maize protoplasts, as previously described (Cao *et al*., 2014). After 16 h incubation at 25 °C, in the dark, GFP signal was observed and photographed using the LSM710 confocal microscope (Zeiss, Germany).

### Maize transformation and plant characterization

The *ZmREM1.3* coding sequence in P178 plants was inserted in the pCAMBIA3301 binary vector so its expression was under the control of the CaMV35S promoter and NOS terminator. The maize Hi‐II (A188 × B73) hybrid (Horn *et al*., 2006) was transformed with the pCAMBIA3301‐ZmREM1.3 vector *via* a previously described *A. tumefaciens*‐mediated transformation protocol (Hensel *et al*., 2009; Van *et al*., 2012). Sixty‐five *T*_0_ plants were recovered, and the *ZmREM1.3*‐containing transformants were identified by a PCR involving *bar* gene‐specific primer (Table S5), genomic DNA and the QuickStix Kit for LibertyLink (Portland, OR). Plants were self‐pollinated, and the *T*_1_ kernels were collected. The *T*_3_ non‐segregating plants were analysed as follows.

The *T*_3_ transgenic and corresponding non‐transgenic lines were inoculated with *P. polysora* spores as described above. For area under the disease progress curve (AUDPC) calculation, disease symptoms were evaluated daily between 8 and 14 days after inoculation. For each evaluated day and plant line, disease severity was calculated as the percentage of infected plants. The disease severity scores were used to calculate

$$\text{AUDPC}=\sum_{i=1}^{n-1}\frac{\left(y_{i}+y_{i+1}\right)\left(t_{i+1}-t_{i}\right)}{2},$$

where *y*_*i*_, disease severity at time *i*,* t*_*i*+1 _− *t*_*i*._

Time (day) interval between two ratings, *n*, number of ratings (Campbell and Madden, 1990; Ji, 2006; Wilcoxson *et al*., 1974). Leaves from five fresh plants were collected at 14 days after inoculation. Three replicates were applied. The collected leaves were quickly frozen in liquid nitrogen and stored at −80 °C until analysed.

To assess the morphological changes induced by a *P. polysora* infection, 1‐μm sections for the same position of leaf tissue 10 cm from the tip of two transgenic (231 and 233) and non‐transgenic sib lines (231sib and 233sib) obtained with a Leica EM UC6 ultramicrotome were stained using periodic acid‐Schiff reagents (Sigma, St. Louis, MO). All samples were observed using the Pannoramic MIDI scanner (Hungary).

### Antibody production and Western blot analysis

To validate the protein levels of ZmREM1.3, rabbit polyclonal antibody was produced. Briefly, the coding sequence of ZmREM1.3 was cloned into pET‐SUMO (Invitrogen, Carlsbad, CA) with SalI and BamHI. The recombinant ZmREM1.3‐His6 fusion protein was purified from Escherichia coli using Ni‐NTA purification system according to the supplier's instructions (Invitrogen). Rabbit polyclonal anti‐ZmREM1.3 was made by immunizing rabbits with the purified ZmREM1.3‐His6 fusion protein (Wuhan GeneCreate Biological Engineering Co., Ltd., Wuhan, China) using the standard WB guaranteed polyclonal antibody protocol (http://www.genecreate.com/m-170.html).

For each maize sample, 15 μg the extracted total proteins were separated on 12% SDS‐PAGE gels and then transferred onto a polyvinylidene difluoride membrane using an electrophoretic transfer system (Bio‐Rad, Hercules, CA). Membranes were blocked for 1 h at room temperature with 5% skim milk in PBST and then probed with an anti‐ZmREM1.3 rabbit polyclonal and anti‐actin mouse monoclonal antibody (Abmart, China) at 4 °C overnight. The membranes were then incubated with horseradish peroxidase (HRP)‐conjugated goat anti‐rabbit IgG or goat anti‐mouse IgG (Boshide, China) for 1 h at room temperature. Immunoreactivity was detected with the HRP‐DAB Detection Kit (Tiangen, China). The intensities of bands were measured by ImageJ (version: k1.45).

### Mutant analysis

Two *ZmREM1.3* UniformMu mutants (mu1084683 in UFMu‐10378 and mu1037985 in UFMu‐03259) were examined for phenotype in response to *P. polysora* infection. UniformMu mutant seeds obtained from the maize stock centre (http://www.uiuc.edu/ph/www/maize) were grown out and self‐pollinated to generate both homozygous mutant lines and corresponding wild‐type lines. A PCR protocol adapted from Settles *et al*. (2004) was used to confirm the presence or absence of insertions. Two markers flanking the insertion were designed. The primers are shown in Table S5. *P. polysora* inoculation and phenotype examination were performed as described above.

### Phylogenetic analysis

The identified ZmREM1.3 and previously classified remorin family proteins (Raffaele *et al*., 2007) were included in a phylogenetic analysis. A phylogenetic tree was constructed as described by Jamann *et al*. (2016).

### Hormone measurement

Endogenous SA and JA levels in transgenic and non‐transgenic plants were measured by high‐performance liquid chromatography (HPLC) and an electrospray ionization MS/MS involving a triple quadrupole mass spectrometer (Pan *et al*., 2010).

### Statistical analysis

All experiments were completed in triplicate. Significant differences (*P *<* *0.05) were identified based on a one‐way analysis of variance completed using the SPSS 17.0 program. Data are presented as the means ± standard error for three replicates.

## Conflict of interest

The authors have declared that no competing interests exist.

## Supporting information


**Figure S1** The frequency distribution of the fold deviation from the mean of each group.

**Figure S2** Kyoto Encyclopedia of Genes and Genomes (KEGG) pathways associated with differentially accumulated proteins.

**Figure S3** Expression level of *ZmREM1.3* in different tissues according to qTeller.

**Figure S4** Confirmation of T_1_ transformants by PCR.

**Figure S5** Southern corn rust resistance identification and ZmREM1.3 expression pattern in Hi‐II parents.

**Figure S6** Molecular identification of the ZmREM1.3 mutants.

**Figure S7** Phylogenetic tree of remorin family proteins in maize.

**Table S1** Peptides identified in resistant and susceptible genotypes.

**Table S2** Proteins identified and quantified by LC‐MS/MS.

**Table S3** Differentially accumulated proteins in the resistant genotype.

**Table S4** Differentially accumulated proteins in the susceptible genotype.

**Table S5** Primers used in this study.

## Data Availability

The mass spectrometry raw data are available via ProteomeXchange (http://www.proteomexchange.org/) with identifier PXD008904.
