# Supplementary figures and images for: Comparative proteomics combined with analyses of transgenic plants reveal ZmREM1.3 mediates maize resistance to southern corn rust
Source: Plant Biotechnol J. 2019 Apr 23;17(11):2153–68. doi: 10.1111/pbi.13129 (PMC6790363; doi:10.1111/pbi.13129)

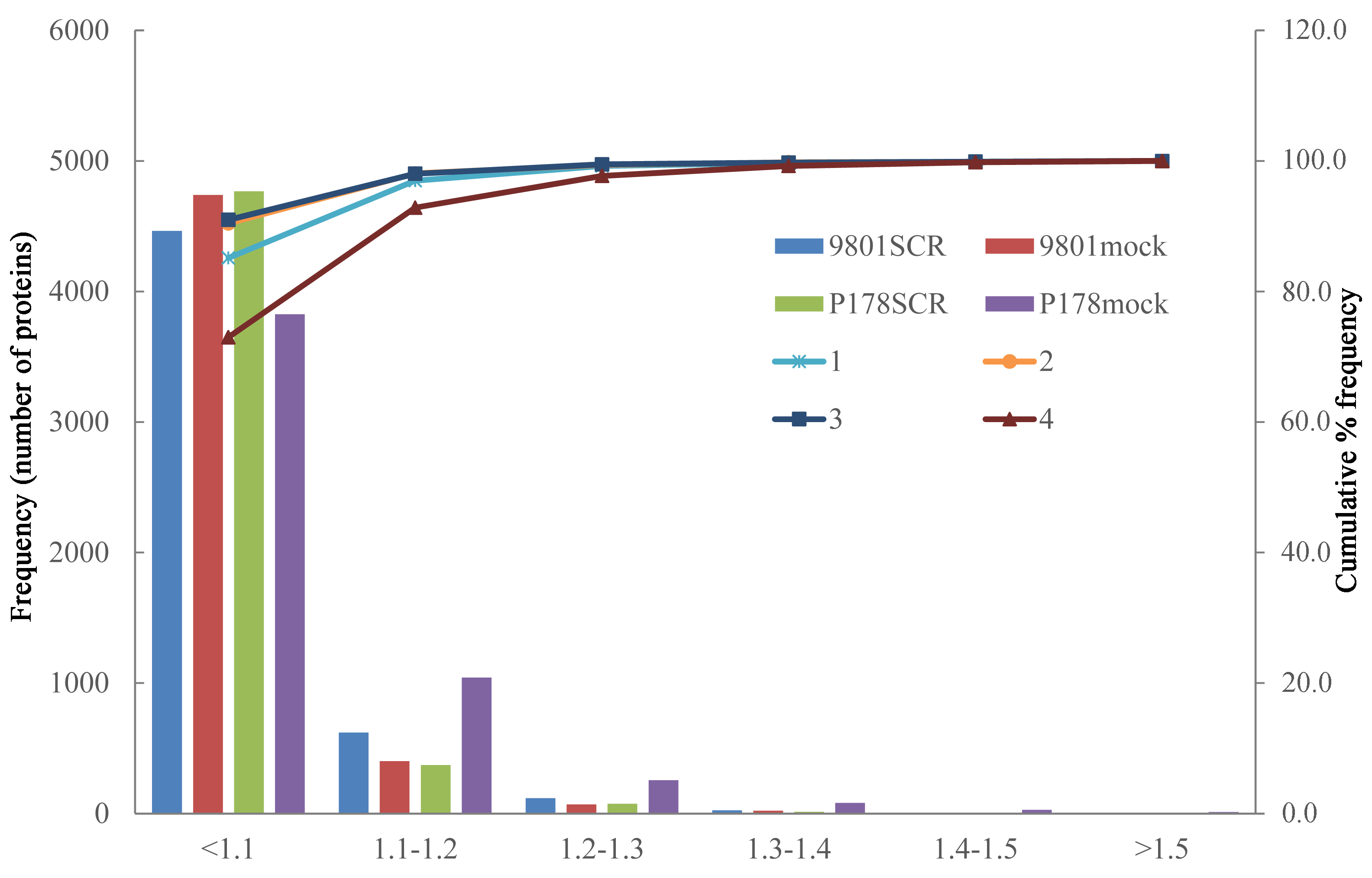

Supplement: Supplementary file 1 — Figure S1 The frequency distribution of the fold deviation from the mean of each group. [file PBI-17-2153-s009.tif]

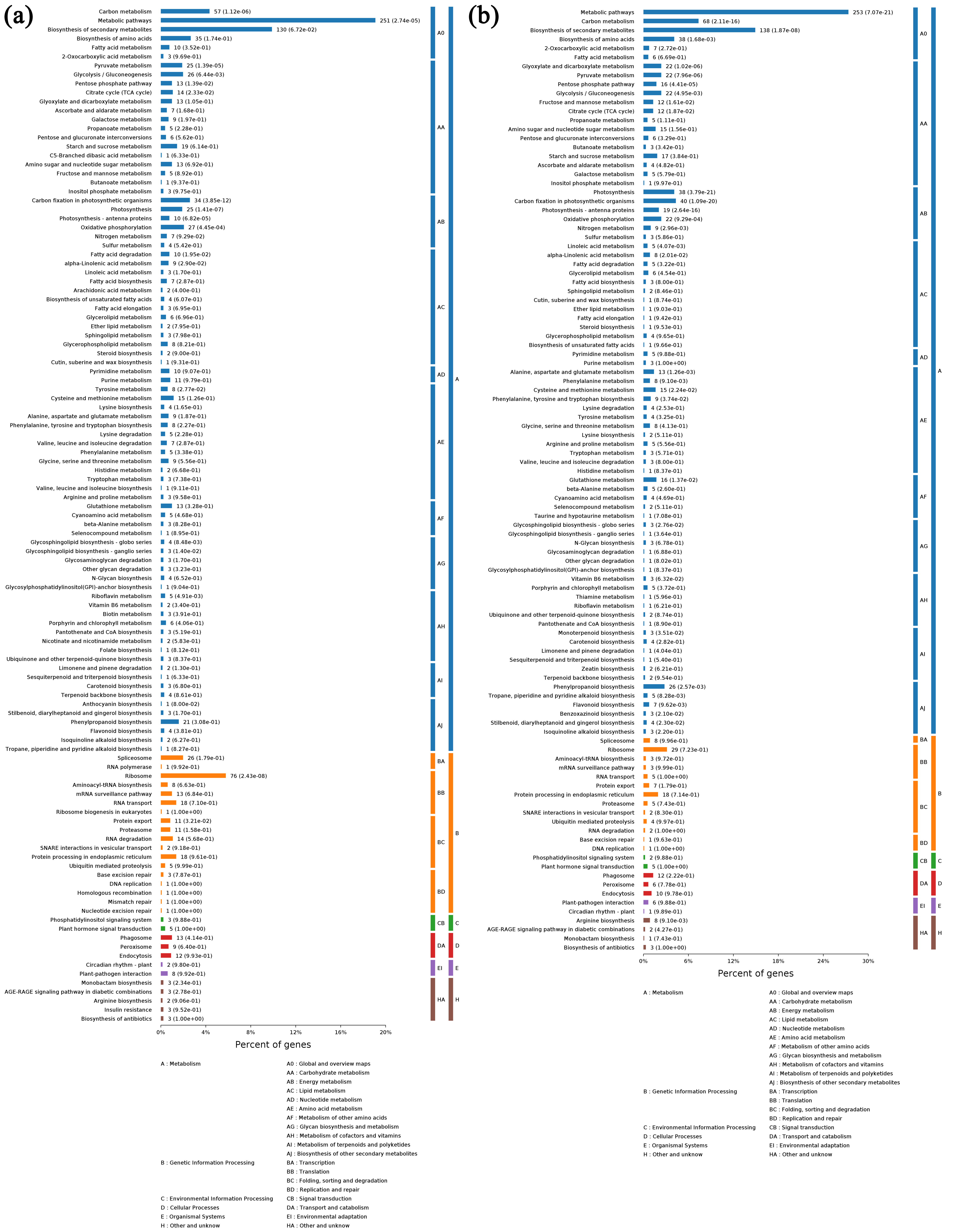

Supplement: Supplementary file 2 — Figure S2 Kyoto Encyclopedia of Genes and Genomes (KEGG) pathways associated with differentially accumulated proteins. [file PBI-17-2153-s008.tif]

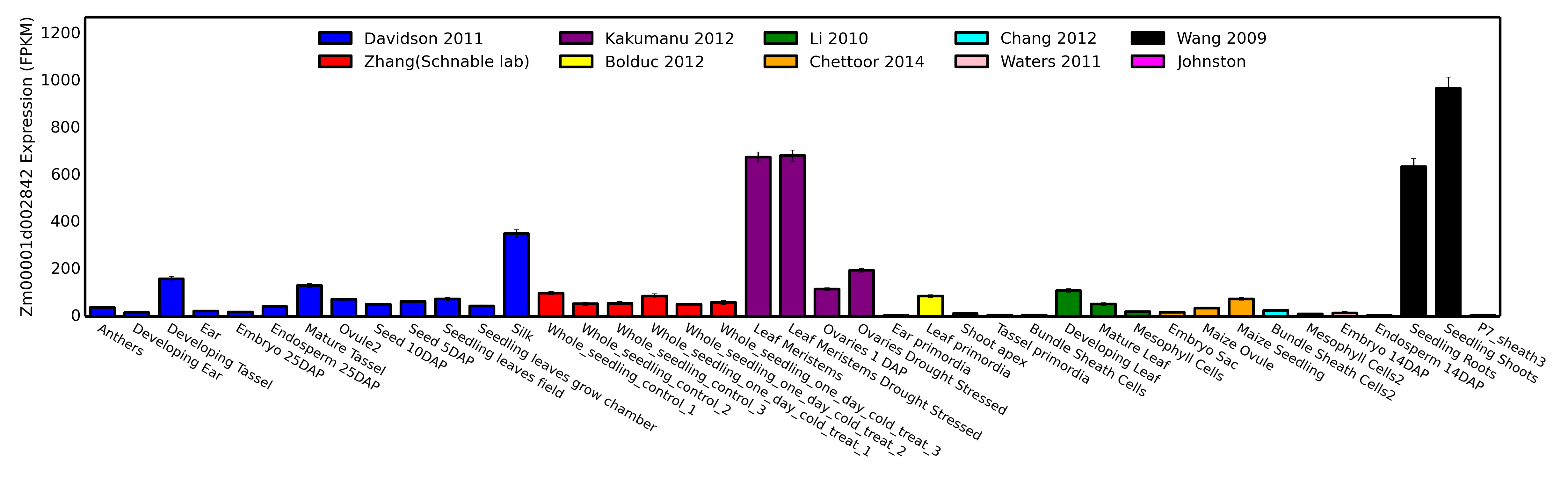

Supplement: Supplementary file 3 — Figure S3 Expression level of ZmREM1.3 in different tissues according to qTeller. [file PBI-17-2153-s007.tif]

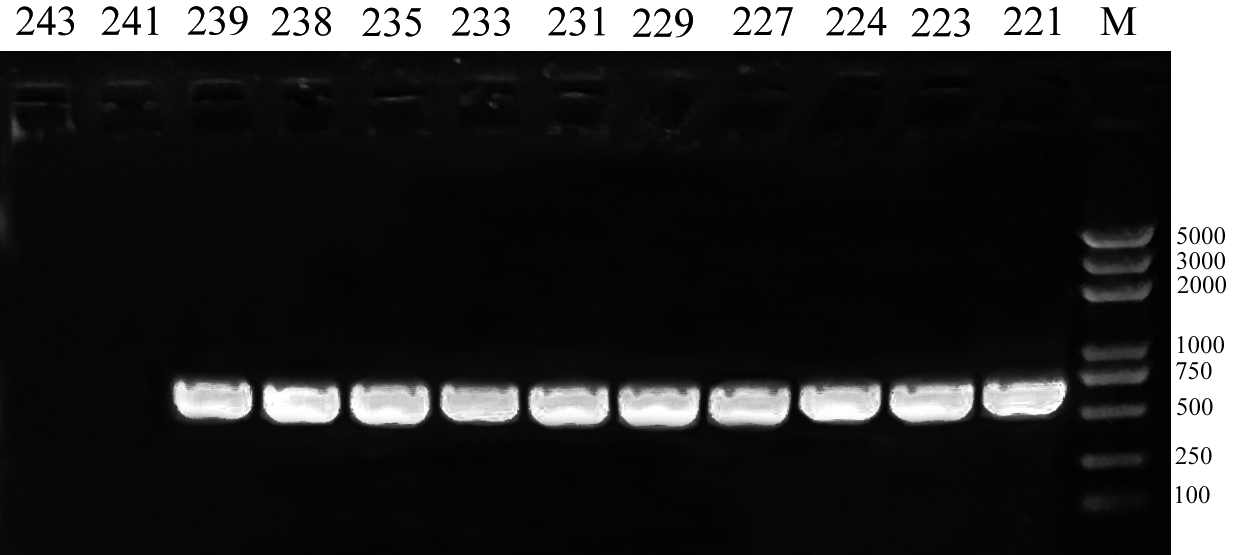

Supplement: Supplementary file 4 — Figure S4 Confirmation of T1 transformants by PCR. [file PBI-17-2153-s011.tif]

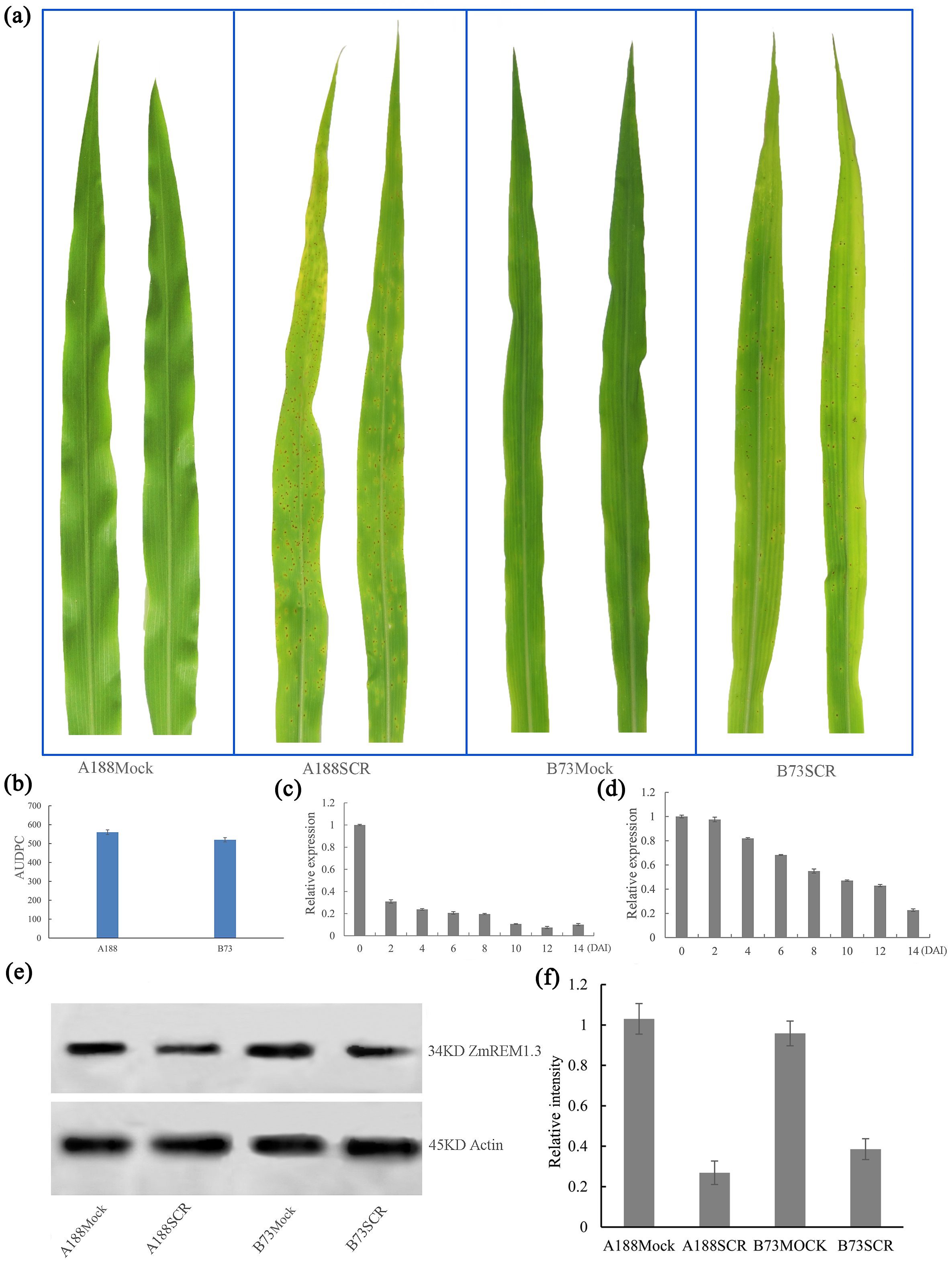

Supplement: Supplementary file 5 — Figure S5 Southern corn rust resistance identification and ZmREM1.3 expression pattern in Hi‐II parents. [file PBI-17-2153-s001.tif]

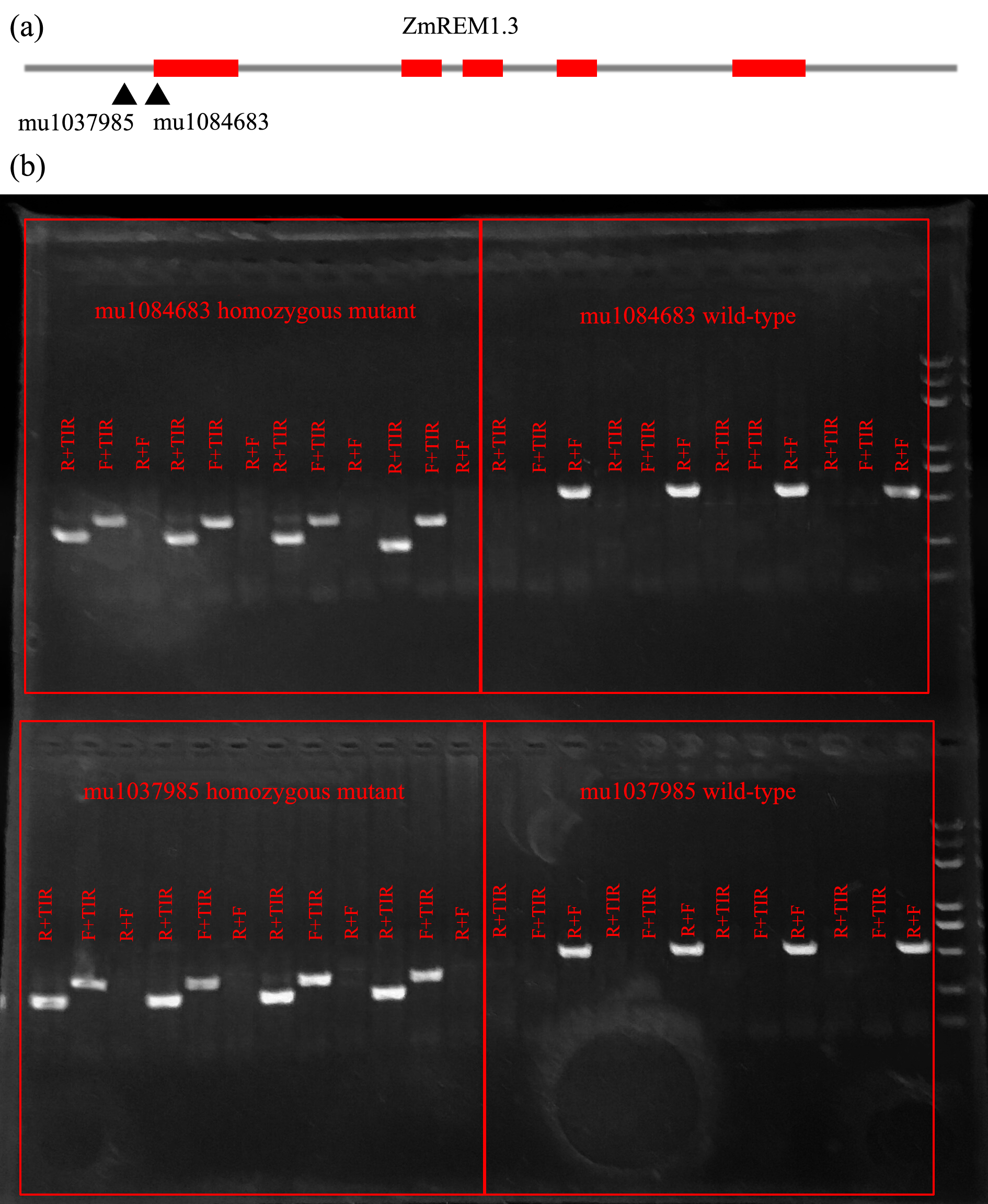

Supplement: Supplementary file 6 — Figure S6 Molecular identification of the ZmREM1.3 mutants. [file PBI-17-2153-s002.tif]

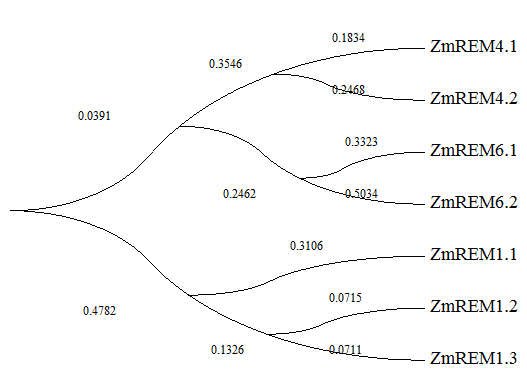

Supplement: Supplementary file 7 — Figure S7 Phylogenetic tree of remorin family proteins in maize. [file PBI-17-2153-s003.tif]
